# Supplementary material for: Predicting visual function by interpreting a neuronal wiring diagram
Source: Nature. 2024 Oct 2;634(8032):113–23. doi: 10.1038/s41586-024-07953-5 (PMC11446822; doi:10.1038/s41586-024-07953-5)
Supplement: Supplementary file 6 — CRF and ERF predictions for individual TmY4 and TmY9 cells. Analogous to Supplementary Data 3, but for TmY target types. Shown are the top four monosynaptic pathways, the strongest pathway passing through each of the top ten intermediary types (ranking from Extended Data Fig. 7), and the trisynaptic pathway Tm1–TmY–Dm3–TmY (see the section entitled Prediction of spatial normalization). [file 41586_2024_7953_MOESM6_ESM.zip › DataS4/TmY9q/720575940632043535.pdf]

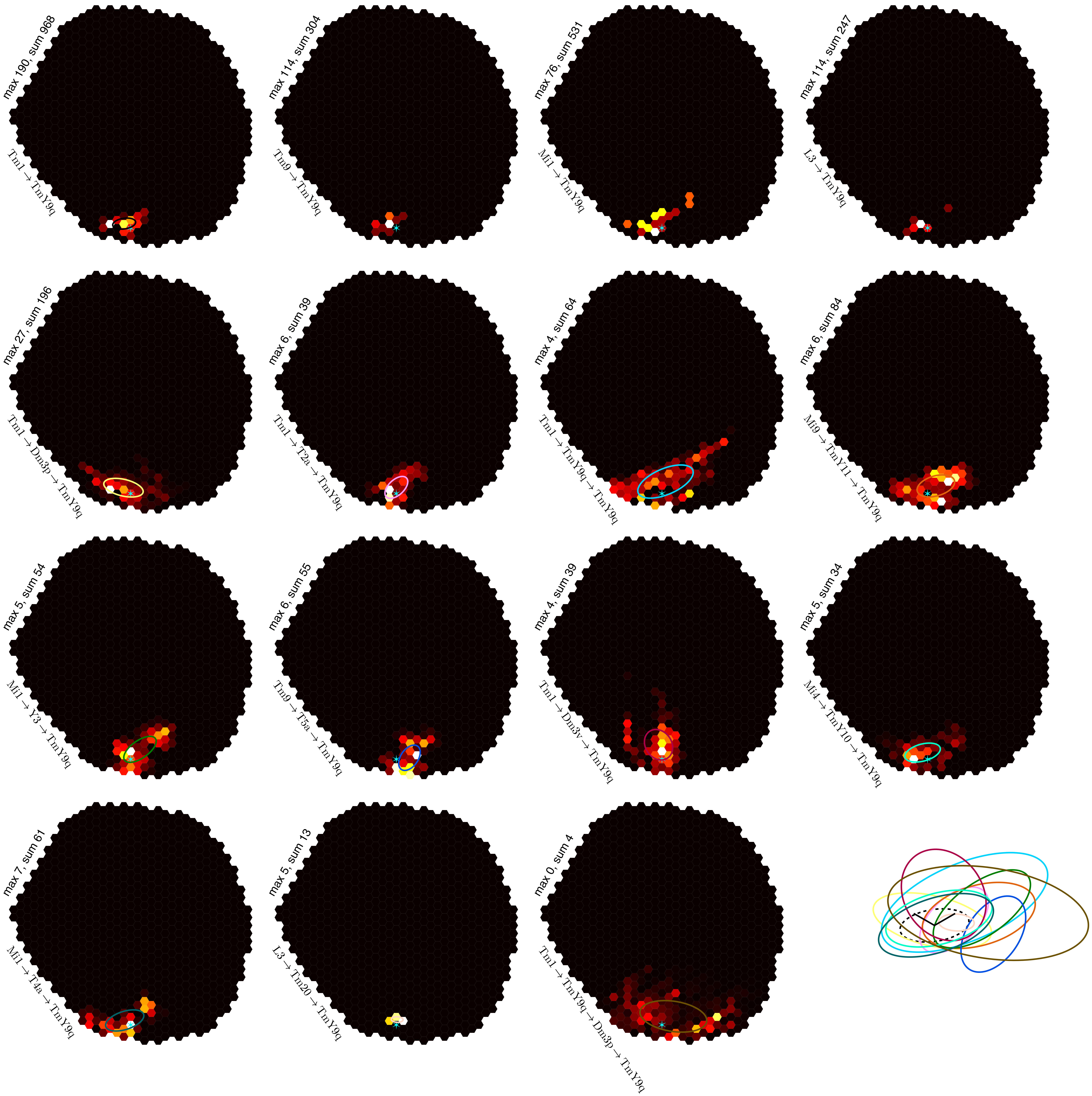

max 190, sum 968
Tm1 → TmY9q
max 114, sum 304
Tm9 → TmY9q
max 76, sum 531
Mi1 → TmY9q
max 114, sum 247
L3 → TmY9q
max 27, sum 196
Tm1 → Dm3p → TmY9q
max 6, sum 39
Tm1 → T2a → TmY9q
max 4, sum 64
Tm1 → TmY9q → TmY9q
max 6, sum 84
Mi9 → TmY11 → TmY9q
max 5, sum 54
Mi1 → Y3 → TmY9q
max 6, sum 55
Tm9 → T5a → TmY9q
max 4, sum 39
Tm1 → Dm3v → TmY9q
max 5, sum 34
Mi4 → TmY10 → TmY9q
max 7, sum 61
Mi1 → T4a → TmY9q
max 5, sum 13
L3 → Tm20 → TmY9q
max 0, sum 4
Tm1 → TmY9q → Dm3p → TmY9q
